# Is early integration of palliative home care in oncology treatment feasible and acceptable for advanced cancer patients and their health care providers? A phase 2 mixed-methods study

**DOI:** 10.1186/s12904-020-00673-3

**Published:** 2020-11-23

**Authors:** Naomi Dhollander, Tinne Smets, Aline De Vleminck, Lore Lapeire, Koen Pardon, Luc Deliens

**Affiliations:** 1 End-of-life Care Research Group, Vrije Universiteit Brussel (VUB) & Ghent University, Corneel Heymanslaan 10, 6K3, room 009, 9000 Brussels, Belgium; 2 Department of Medical Oncology, Ghent University Hospital, Ghent, Belgium; 3 Cancer Research Institute Ghent (CRIG), Ghent University Hospital, Ghent, Belgium; 4 Department of Public Health and Primary Care, Ghent University Hospital, Ghent, Belgium

## Abstract

**Background:**

To support the early integration of palliative home care (PHC) in cancer treatment, we developed the EPHECT intervention and pilot tested it with 30 advanced cancer patients in Belgium using a pre post design with no control group. We aim to determine the feasibility, acceptability and perceived effectiveness of the EPHECT intervention.

**Methods:**

Interviews with patients (*n* = 16 of which 11 dyadic with family caregivers), oncologists and GPs (*n* = 11) and a focus group with the PHC team. We further analyzed the study materials and logbooks of the PHC team (*n* = 8). Preliminary effectiveness was assessed with questionnaires EORTC QLQ C-30, HADS and FAMCARE and were filled in at baseline and 12, 18 and 24 weeks.

**Results:**

In the interviews after the intervention period, patients reported feelings of safety and control and an optimized quality of life. The PHC team could focus on more than symptom management because they were introduced earlier in the trajectory of the patient. Telephone-based contact appeared to be insufficient to support interprofessional collaboration. Furthermore, some family caregivers reported that the nurse of the PHC team was focused little on them.

**Conclusion:**

Nurses of PHC teams are able to deliver early palliative care to advanced cancer patients. However, more attention needs to be given to family caregivers as caregiver and client. Furthermore, the home visits by the PHC team have to be further evaluated and adapted. Lastly, professionals have to find a more efficient way to discuss future care.

## Background

The World Health Organization (WHO) advises to provide palliative care early in the course of a life-threatening illness – i.e. from diagnosis of advanced cancer on –, in conjunction with other therapies that are intended to prolong life [1]. Similarly, the American Society of Clinical Oncology (ASCO) and the Multinational Association of Supportive Care in Cancer (MASCC), the European Society of Medical Oncology (ESMO) and the European Association of Palliative Care (EAPC) in Europe also recommend palliative care to be introduced early in the disease trajectory concurrent with active treatment for advanced cancer patients with a life expectancy of 6 to 24 months [2].

Over the past few years, a growing number of studies have investigated the effects of integrating palliative care early in oncology care on the quality of life of patients [3–10]. Most studies showed a positive effect, but all focused on integrating early palliative care in cancer treatment in the hospital or in the outpatient setting. Up till now, no studies have been published on integrating palliative care early at home. Palliative care is indeed not only provided in hospitals or via outpatient clinics but also and especially at home. A recent systematic literature review shows that the majority of cancer patients prefers to die in their own homes [11] and palliative home care allows people to stay at home until death, supported and surrounded by those close to them [12]. However, integrating palliative *home* care into standard oncological treatment is more complex than early integration in hospital or outpatient clinics, as it requires interprofessional and transmural collaboration – i.e. collaboration between multidisciplinary teams at home and in hospital. Hence, there is a need for an evidence-based model for the early integration of palliative home care in the current regular cancer treatment for advanced cancer patients that takes account of these complexities.

To facilitate the early integration of palliative *home* care in cancer treatment, we developed the Early Palliative Home Care Embedded in Cancer Treatment intervention (hereinafter – EPHECT intervention) (Table 1) [13]. Because the EPHECT intervention is one of the first interventions worldwide focusing on the early integration of palliative home care in oncology care in the hospital, it is important to first evaluate the feasibility, acceptability and preliminary effectiveness of this intervention in a phase 2 study before testing its effectiveness in a large-scale phase 3 randomized controlled trial (RCT). In this paper, we present the results of our phase 2 study providing essential information about the strengths and weaknesses of this specific intervention, and insights about how an existing multidisciplinary service of palliative home care can be optimally introduced and integrated early in a trajectory of advanced cancer.

**Table 1:** Overview of the components of the EPHECT intervention

Component	Description
Education of involved professionals of the palliative home care team	- Educational session of two hours for members of the PHC team consisting of group discussions, case studies and education on drug therapies and side effects - Training for the PHC team in working with the intervention materials
Information of involved oncologists	- Involved oncologists are informed about the intervention and the role of the PHC team
General practitioner (GP) as coordinator of care	- GP were contacted by data nurse to give permission for introducing PHC to his/her patient - GP then contacted the PHC team to plan the first visit - GP is the central coordinator of care and communicates with the PHC team and oncologist
Regular home visits by the nurse of the palliative home care (PHC) team	- In-person home visits with patient and family caregiver - Recommendation of minimum one home visit per month, but to be discussed with patient in first consultation - Consultations supplemented with in-between telephone contacts if needed
Semi-structured home visits not only focusing on symptom management, but also on psychological and social care	- Semi-structured conversation guide used in home visits of the PHC team in which following topics are embedded: o Understanding and perception of illness o Routine symptom management (ESAS at each visit) o Organization of care o Coping mechanisms o Quality of life of patient and family caregiver o Preferences for future care
Interprofessional and transmural collaboration	- Collaboration and communication via telephone contacts - Patients discussed by the PHC team during weekly meetings - GP should be contacted after each home visit and if needed after weekly meeting - If needed, GP should contact oncologist to discuss further actions

The aims of this study are [1] to assess the feasibility and acceptability of the study procedures (i.e. recruitment, inclusion criteria) [2] to assess the feasibility and acceptability of the components of the EPHECT intervention, according to patients, family caregivers and healthcare professionals; and [3] to explore preliminary effectiveness and perceived effects of the EPHECT intervention on the quality of life of patients and family caregivers.

## Methods

### Study design

We designed a phase II pre-post trial following the guidance of the Medical Research Council for the development and evaluation of complex interventions [14, 15]. To assess feasibility and acceptability of the intervention components, we conducted interviews with patients, family caregivers, general practitioners (GPs) and oncologists and a focus group with the palliative home care (PHC) team after the intervention. Patients and family caregivers also had to fill in questionnaires at baseline and at 12, 18 and 24 weeks follow-up to evaluate the preliminary effectiveness of the intervention. Preliminary effectiveness was assessed in order to evaluate the feasibility and acceptability of the questionnaires used and to select primary and secondary outcomes for a follow-up Phase 3 RCT.

After oncologists screened patients for their eligibility for the study (see Table 2 for inclusion criteria), the data nurse introduced the EPHECT intervention to all eligible patients and asked whether they were willing to participate. When patients agreed to participate and filled in an informed consent, the data nurse contacted their GPs because in Belgium the GP has to give permission to the PHC team to start a trajectory with a patient. More details about the content and development of the EPHECT intervention can be found in Table 1 and elsewhere [13].

**Table 2:** Inclusion and exclusion criteria

Inclusion	Exclusion
- Non-curative treatable solid cancer diagnosis - Life expectancy of 6 to 24 months (assessed by treating oncologist) - Identification as having palliative needs (assessed by treating oncologist) - Active anticancer treatment - 18 years or older - Patients with the ability to read and respond to questions in Dutch	- Hematological malignancy as primary diagnosis - Not housed in the Brussels region - No active anticancer treatment - No permission of the GP - More than one palliative care consultation with palliative team in hospital before inclusion - Involved in another palliative care intervention study - Impaired cognition

Similar to previous models of early palliative care in the hospital, the EPHECT intervention consists of regular visits of the palliative care team to the patient, in this case at home, supported by a semi-structured conversation guide focusing on symptom management, psychological and social care. The EPHECT intervention also incorporates a structured procedure by telephone contact (see Table 1) for collaboration between the health care settings, a component that is more complex and elaborated compared with the other existing models as integrating palliative home care requires collaboration between settings of home (self-care and family caregivers), primary care (GPs) and the hospital care (oncologists, oncology nurses, etc.).

We used the Consolidated Standards of Reporting Trials (CONSORT) extension for Pilot and f-Feasibility Trials Checklist as a methodological guidance for reporting the EPHECT intervention [16].

### Settings and participants

This study was performed in the home setting of advanced cancer patients in the Brussels region. We recruited patients with advanced cancer from the Medical Oncology departments of a university hospital and a general hospital. All patients in treatment and all newly diagnosed patients with a solid cancer diagnosis were screened in the hospitals by the oncologists for their eligibility (*n* = 41) (Table 2).

The Ethics Committees of the University Hospital and of the regional hospital approved the study protocol. All patients, informal caregivers and GPs involved in the study provided their written informed consent.

#### Data collection

An overview of the methods to assess the feasibility, acceptability and preliminary effectiveness of the EPHECT intervention can be found in Table 3.

- *Feasibility and acceptability of the study procedures* was assessed by recording the number of all eligible patients (and reasons for being not eligible), patients who were asked to participate in the study, and patients who agreed to participate (and reasons for non-participation). We also recorded drop out during the study (and reasons for drop out), and time of death. Information about time of death was collected from medical records and information about the recruiting procedure was kept in the logbooks of the researcher and the data manager.
- *Feasibility of the intervention* was assessed quantitatively, by registering the number and duration of the visits done by the PHC team. The content of the conversations and the estimated time spent on the topics described in the semi-structured conversation guide were collected by the nurses of the PHC team in the electronic patient file. They also registered the amount of and reasons for contact of the PHC team with other professional caregivers in the logbook for transmural collaboration. Nurses of the PHC team were asked to keep a record of contacts with GPs and oncologists in the care for a patient. The contacts between GPs and oncologists were evaluated in the semi-structured interviews with GPs and oncologists.
- *Acceptability of the intervention* was assessed with qualitative methods, i.e. semi-structured interviews with patients (*n* = 16, of which 11 were dyadic interviews with a family caregiver), GPs and oncologists (*n* = 11) involved in the intervention. Interviews with patients, family caregivers and GPs focused on perceived strengths, concerns and weaknesses of the intervention and whether the intervention was acceptable. Interviews with oncologists focused on exploring strengths and weaknesses of the intervention, and on their own role within the intervention including their reflection on the inclusion criteria. We also conducted a focus group with the PHC team (*n* = 8) involved in the intervention, focusing on their experiences with the intervention and the usability of the intervention materials.
- The *preliminary effectiveness of the intervention* was assessed with questionnaires for patients and family caregivers by comparing patient and caregiver outcomes at baseline and after 12, 18 and 24 weeks. The primary objective of the preliminary effectiveness assessment was the patient’s quality of life measured with the European Organisation for Research and Treatment of Cancer Quality of Life Questionnaire (EORTC QLQ C-30) [17]. Secondary objectives were [1] the patient’s mood, assessed with the Hospital Anxiety and Depression Scale (HADS) [18] and illness understanding, measured by a questionnaire developed by Temel et al. [5] and translated by Vanbutsele et al. [19], [2] the informal caregivers’ mood (HADS), satisfaction with care, assessed with the Family Satisfaction with End-of-Life Care (FAMCARE) [20] and illness understanding. Those assessments were done to evaluate the feasibility and acceptability of the questionnaires used and to select primary and secondary outcomes for a follow-up Phase 3 RCT.
- We also assessed *perceived effectiveness of the intervention* in the interviews with patients, family caregivers, GPs, oncologists and the focus group with the PHC team.

**Table 3:** Overview of methods to assess feasibility, acceptability and perceived effectiveness of the EPHECT intervention

MEASURES	METHOD	INDICATORS
**Feasibility study procedures**	Logbooks researcher & data nurse Electronic patient file	Inclusion procedure Drop out & time of death Questionnaires • Missings • Responses at baseline and follow-up
**Feasibility & acceptability intervention**	Quantitative: • Logbooks Omega • Record of time spent on topics of the semi-structured guide in electronic patient file • Record of interprofessional contact in electronic patient file Qualitative: • Interviews with patients and family caregivers, GPs and oncologists • Focus group with PHC team	Quantitative: • Amount and content of visits • Time spent on topics of the semi-structured guide • Interprofessional contact Qualitative: • Acceptability of the intervention components • Suggestions for improvement
**Preliminary and perceived effectiveness**	Quantitative: • Questionnaires filled in by patients and family caregivers at baseline and follow-up at 12, 18 and 24 weeks Qualitative: • Interviews with patients and family caregivers	Quantitative: • Patient o Quality of life (EORTC QLQ C-30) o Mood (HADS) o Disease insight • Family caregiver o Satisfaction with care (FAMCARE) o Mood (HADS) o Disease insight Qualitative: • Patients’ and family caregivers’ perceived effects of the EPHECT intervention

#### Data analysis

##### Qualitative data

A topic guide for the interviews and the focus group, consisting of open questions and a set of prompts, was developed by a member of the research team (ND) and reviewed within a multidisciplinary team of researchers. Interviewers were a member of the research team (ND) and a data manager (FS). The interview guide consisted of questions regarding the feasibility and acceptability of the intervention components and the perceived effects of the intervention: 1) What are your experiences with the intervention and the intervention components?; 2) What are the barriers and facilitators for implementing the intervention into clinical practice?; 3) Did the intervention had effect on your personal life (patients and family caregivers) or your professional life (professional caregivers)?.

All interviews and the focus group were audio-recorded, transcribed verbatim and analysed by a junior and a senior researcher (ND and TS). Interviews were analysed using thematic content analysis [21]. The data analysis was both inductive and deductive. First, we searched for preliminary codes on the basis of the underlying structure of the interview. Second, those codes were deductively linked to the intervention components. The two researchers compared their codes and when discrepancies occurred, consensus was sought within the multidisciplinary research team.

Qualitative data analysis software (NVivo 11) was used.

##### Quantitative data

We included all data collected during the study period. We calculated mean and standard deviations for each questionnaire at t0, t1, t2 and t3. The mixed-effects linear model for repeated measures represents a proper statistical method to assess possible changes in scores over time, allowing for differing numbers of measures per patient and accounting for missing values, by incorporation of all available data into a single model spanning the entire follow-up period. These characteristics make this model ideal for investigating the changes over time.

## Results

### Feasibility and acceptability of the study procedures

#### Inclusion & drop out of patients

**Fig. 1:**
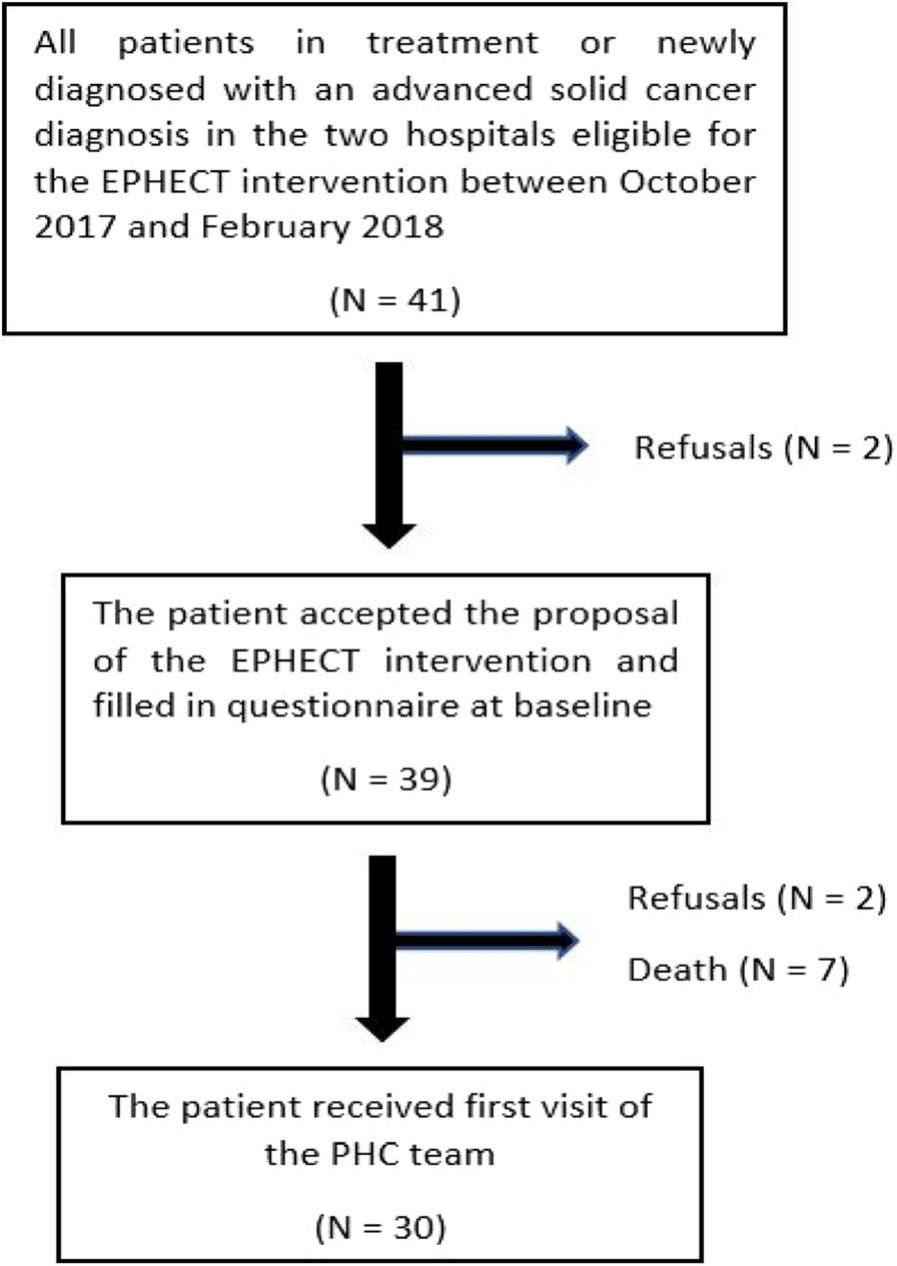
Flowchart of the study

From October 2017 to February 2018, all patients with advanced cancer receiving active oncological treatment in the two involved hospitals were screened for eligibility by all oncologists. The EPHECT intervention was introduced by the data manager to 41 eligible patients of which 39 consented to participate and filled in a questionnaire at baseline. Of those 39 patients, 7 patients died and 2 dropped out between baseline measurement and the first visit of the PHC team four weeks later. The 2 patients who dropped out were not convinced of the added value of the intervention. A flowchart of the study can be found in Fig. 1.

Thus, 30 of the 41 eligible patients (73%) were visited at least once by the PHC team. Characteristics of those 30 patients are presented in Table 4. The most common cancer type was digestive cancer (47%), followed by lung (17%) and Triple Negative breast cancer (17%). 26/30 patients (86,6%) filled in the questionnaire at 12 weeks, 21/30 (70%) at 18 weeks and 18/30 (60%) at 24 weeks. Most of the patients who dropped out during the 24 weeks died, 2 patients dropped out due to personal reasons.

**Table 4:** Patient characteristics at baseline (*N* = 30)

	N	%
Age		
18–65	15	50%
> 65	15	50%
Gender		
Male	18	60%
Female	12	40%
Living situation		
Home (cohabiting)	26	86,7%
Home (alone)	4	13,3%
Partner		
Yes	27	90%
No	3	10%
Highest level of education		
Lower than high school	3	10%
Lower level in high school	11	36,7%
Higher level in high school	10	33,3%
College, university	6	20%
Primary cancer diagnosis		
Digestive	14	46,7%
Breast cancer triple negative	5	16,7%
Lung	5	16,7%
Gynecological	3	10%
Sarcomas	1	3,3%
Head-neck	1	3,3%
Prostate	1	3,3%

Of those 30 patients, 13 included a family caregiver who gave consent and also filled in a questionnaire at baseline, 12, 18 and 24 weeks. Drop out in family caregivers during the intervention period was directly linked to drop out in patients.

#### Feasibility of inclusion and exclusion criteria

All 5 oncologists reported in the interviews that it was easier to estimate a life expectancy of six months than two years. Instead of using prognosis as primary inclusion criterium, oncologists suggested to introduce palliative home care from diagnosis of an advanced disease. Two made an exception for breast cancer because those patients could live for five or ten years with metastases. They suggested to introduce palliative home care to those patients after two episodes of chemotherapy. Some patients mentioned that they would have liked palliative home care to be introduced even earlier in the disease trajectory because it would have created more time and space for building up a relationship with the PHC nurse.

### Feasibility and acceptability of the intervention components

To evaluate the feasibility and acceptability of the intervention components, information from the logbooks of professional caregivers, conversation guides filled in by the PHC team and electronic patient files were combined with information gathered from the interviews with patients, family caregivers and professional caregivers. Because the feasibility and acceptability of the intervention are closely linked, we have decided to discuss each intervention component separately with a combination of both the quantitative and the qualitative data.

Only one GP was suspicious in the beginning about the added value of visits by the PHC team for his patient. However, all GPs eventually gave permission to include their patients in the EPHECT intervention.

#### Education for nurses of the PHC team

Although nurses of the PHC team had the possibility to ask questions about oncological diseases and treatments in the information sessions, they had the feeling in the first home visits that they did not know enough to answer questions about oncology care. Instead of answering these questions, they empowered the patients in asking the questions to the oncologist on the first following consultation. Despite nurses’ feelings of uncertainty about their ability to handle issues related to diagnoses, prognoses and oncological treatments at the beginning of the intervention period, interviews with patients and family caregivers showed that the nurse of the PHC team often provided information about diagnosis and prognosis if needed and the nurses confirmed that experience with early palliative care trajectories was important in increasing their self-efficacy.

#### Regular home visits by the PHC team

During the six-month intervention period, nurses of the PHC team visited the patients on average four times. Six patients were visited only once, five of them died before the second visit and one patient found the visits too time-consuming and not useful. Ten of 30 patients were visited monthly as recommended in the intervention protocol, resulting in six or more visits. Reasons that members of the PHC team gave for visiting patients less often or not monthly were that sometimes patients were admitted to the hospital and visits had to be rescheduled to a later time. Some visits to the patient’s home were replaced by a telephone call when the patient did not need a home visit for instance when they stabilized or their condition improved. More frequent visits (more often than once a month) occurred when the patient’s health condition got worse or when the patient was closer to death.

The 15 interviews with patients and family caregivers showed that they did not have problems with being involved in palliative home care whilst receiving ongoing anticancer treatment and that visits were considered necessary monthly in the beginning of the trajectory for building a trusting relationship. Most patients found that once this relationship was established, monthly visits were acceptable but not needed and most patients were glad that the visits were planned by the PHC team. Some patients told that they would not contact the PHC nurse by themselves even though they evaluated the visits as beneficial. The main reason for this lack of taking initiative was that patients had other things to worry about than contacting the PHC nurse. If their health condition would stabilize or improve, some patients in that case recommended to plan PHC visits according to their needs. In the focus group the PHC team agreed for future care on planning the visits in dialogue with the patient and the family caregiver, whilst following them up by telephone.

#### Semi-structured conversation guide

Most nurses evaluated the conversation guide as a useful tool to structure the conversations with the patient during the home visits and to reflect back with the patient and family caregiver on what was discussed during previous home visits. Few patients noticed that the visits were always structured in the same way, but it did not bother them.

Analyses of the patient files show that the main focus of the visits was on physical symptom management. Some patients reported that the nurse of the PHC team had given them and the GP advise on medication use. Analyses of the patient files also showed that being involved earlier gave the nurses of the PHC team the opportunity to provide holistic care and to spend more time on topics other than symptom management like disease insight – reflecting on what the patient knows about his/her disease, treatment and prognosis –, coping with the illness and the treatments and advance care planning. Members of the PHC team revealed in the focus group that they were initially concerned that providing more holistic care would require a different approach and different skills, but the more experience they gained with the intervention, the more they felt it was their responsibility to discuss not only physical symptoms, but also preferences for future care, coping or other psychosocial issues. According to patients and to the nurses of the PHC team, psychosocial and existential issues were indeed also important topics in the home visits with the patient. One patient received support and advise for sexual problems, others reported support on financial and practical level. The PHC team also talked with the patients about diagnoses and prognoses, information that was often not given by the oncologist.

Lastly, analyses of the interviews showed that during the home visits, little attention was given to how the family caregiver was coping. Although most family caregivers reported that they had received enough support of the PHC team, some mentioned that the nurse of the PHC team was mainly focused on the patient and that they had expected to be involved more.

*“I’d expected more from the support of that nurse. The focus was still on my partner and I still had the feeling that nobody listened to my concerns.” (family caregiver)*

#### Interprofessional and transmural collaboration with the GP as coordinator of care

Analyses of the logbooks of the PHC team show that for most patients, the GP was only contacted once by a nurse of the PHC team, namely when palliative home care was initiated, to let the GP know that the PHC team was introduced to the patient. This contact was always by telephone. Only for a few patients the GP was contacted more regularly, mostly when the patient deteriorated and was in the terminal phase of life or when medication had to be adjusted. GPs therefore said that it was difficult to take up their responsibility in being the coordinator of the care trajectory. The PHC team rarely contacted the oncologist directly because they did not want to pass-by the GP.

Interviews with GPs and oncologists and the focus group with the PHC team showed that PHC teams evaluated contact with the GP as unneeded when a patient was stable, whilst GPs reported that they would have liked to receive a short report after each visit from the PHC team. The interviews also revealed that GPs as well as nurses of the PHC team were sometimes difficult to reach by telephone resulting in suboptimal interprofessional communication and collaboration. Furthermore, the majority of the GPs mentioned in the interviews that they preferred being the communicator between the PHC team and the oncologist, whilst all oncologists preferred to receive information directly from nurses of the PHC team so that they could react immediately.

Based on the feasibility and acceptability of the intervention components, we made suggested changes to the EPHECT intervention which are shown in Table 5.

**Table 5:** Suggested changes to the EPHECT intervention

Component	Description	Acceptability and feasibility	Suggested changes to the intervention
Education for involved professionals	- Educational session of two hours for members of the PHC team consisting of group discussions, case studies and education on drug therapies and side effects - Training for the PHC team in working with the intervention materials - Involved oncologists will be informed about the intervention and the role of the PHC team	- Training was too short to make nurses of the PHC team comfortable in having discussions on oncology care	- Ongoing educational sessions or possibilities to contact oncologists if questions about oncology care arise. - Educational sessions should focus more on involving the family caregiver.
General practitioner (GP) as coordinator of care	- GP will be contacted by data nurse to give permission for introducing PHC to his/her patient - GP will then contact the PHC team to plan the first visit - GP is the central coordinator of care and communicates with the PHC team and oncologist	- GP was rarely contacted by the PHC team, because the nurses of the PHC team thought it was not needed to contact the GP if the patient was stable. - GPs reported difficulties in taking up the role of coordinator of care because they were not involved more than in standard care.	- Clear agreements have to be made about how and when communication has to take place, in dialogue with the involved GPs, PHC team and oncologists.
Regular home visits by the palliative home care (PHC) team	- In-person consultations with patient and family caregiver - Recommendation of minimum one home visit per month, but to be discussed with patient in first consultation - Consultations can be supplemented with in-between telephone contacts if needed	- Patients, family caregivers and nurses of the PHC team said that it was necessary to install monthly visits in the beginning of the trajectory to build up a relationship. - Once the relationship is built, monthly visits are not needed as long as the situation is stable and visits should be planned according to the needs of the patients and family caregivers.	- Monthly consultations at the beginning of the trajectory. - Later on: visits need to be planned according to the needs of patients and family caregivers. - Regular follow-up by telephone on initiative of the PHC team.
Semi-structured contacts not only focusing on symptom management, but also on psychological and social care	- Semi-structured conversation guide to be used in home visits of the PHC team in which following topics are embedded: o Understanding and perception of illness o Routine symptom management (ESAS at each visit) o Organization of care o Coping mechanisms o Quality of life of patient and family caregiver o Preferences for future care	- Being involved earlier provided nurses of the PHC team time to not only focus on symptom management, but also on other core domains of palliative care as recommended in the semi-structured conversation guide. - Coping of the family caregiver was the topic least discussed and some family caregivers reported in the interviews that they had the feeling during the intervention that the nurse of the PHC team was mainly focused on the patient.	- More attention needs to be given to the family caregiver in the home visits.
Interprofessional and transmural collaboration	- Collaboration and communication via telephone contacts - Patients will be discussed by the PHC team during weekly meetings - GP will be contacted after each home visit and if needed after weekly meeting - If needed, GP will contact oncologist to discuss further actions	- Telephone-based contact was insufficient. - GPs and nurses of the PHC team had different opinions about when contact was needed. - GPs wanted to be the communicator between the PHC team and the oncologist, but were rarely contacted. Oncologists reported to prefer direct contact with the nurses of the PHC team.	- Face-to-face contact between all professional caregivers needs to be installed to discuss future care. - Clear agreements have to be made about how and when communication has to take place, in dialogue with the involved GPs, PHC team and oncologists.

### Preliminary effectiveness of the EPHECT intervention

Table 6 and Table 7 show results of preliminary effectiveness of the EPHECT intervention on quality of life, mood and satisfaction with care. No significant deterioration nor improvement over time was observed for any questionnaire scales and subscales. Initial scores of family caregivers on the anxiety scale were high and remained high during the intervention.

**Table 6:** Evolution of mean scores over time in quality of life and mood of patients with advanced cancer at baseline, 12, 18 and 24 weeks

Patients’ outcomes	Range	Assessment				
		Baseline (t0)	12 weeks (t1)	18 weeks (t2)	24 weeks (t3)	*P value*
		(N = 30)	(*N* = 26)	(*N* = 22)	(*N* = 18)	
		Mean ± SD	Mean ± SD	Mean ± SD	Mean ± SD	
**EORTC QLQ C-30**						
Global QOL	0–100	60,5 ± 16	58 ± 17,7	61,9 ± 17,6	62,5 ± 17,2	0,81
Physical functioning	0–100	60,9 ± 23,8	60,5 ± 23,2	60,9 ± 21,2	65,4 ± 23,1	0,90
Role functioning	0–100	59,4 ± 31,5	51,3 ± 29,6	53 ± 28	55,6 ± 32,8	0,78
Emotional functioning	0–100	69,6 ± 18,3	66 ± 24,6	67 ± 23,4	70,8 ± 24	0,88
Cognitive functioning	0–100	75 ± 23,1	68 ± 25,4	75 ± 25,1	69,4 ± 29,8	0,67
Social functioning	0–100	72,2 ± 23	73,1 ± 20,6	75,8 ± 19,1	69,4 ± 29,8	0,86
**HADS**						
HADS – anxiety	0–21	7,9 ± 4,1	7 ± 4	6,2 ± 4,3	7,5 ± 5,1	0,55
HADS – depression	0–21	7,4 ± 3,6	7,3 ± 4,3	6,6 ± 3,7	7,1 ± 4,5	0,90

EORTC QLQ C-30: European Organisation for Research and Treatment of Cancer Quality of Life Questionnaire (for all the EORTC QLQ C-30 scales the higher the score, the better quality of life); *HADS* Hospital Anxiety and Depression Scale (for both HADS subscales the higher the score, the higher the distress)

**Table 7:** Evolution of mean scores over time in satisfaction with care and mood of family caregivers at baseline, 12, 18 and 24 weeks

Caregiver outcomes	Range	Assessment				
		Baseline (t0)	12 weeks (t1)	18 weeks (t2)	24 weeks (t3)	*P value*
		(*N* = 13)	(*N* = 12)	(*N* = 9)	(*N* = 7)	
		Mean ± SD	Mean ± SD	Mean ± SD	Mean ± SD	
**FAMCARE**						
Total score	0–100	70,7 ± 9,9	70,5 ± 13,7	70,6 ± 12,9	71,4 ± 17,5	0,99
**HADS**						
HADS – anxiety	0–21	10,5 ± 4,5	10,5 ± 4,6	10,3 ± 3,7	11,3 ± 6,3	0,98
HADS – depression	0–21	8,5 ± 2,5	8,2 ± 4,4	6,6 ± 4,5	7,3 ± 5	0,73

FAMCARE: Family Satisfaction with End-of-Life Care (higher score means higher satisfaction with care); HADS: Hospital Anxiety and Depression Scale (for both HADS subscales the higher the score, the higher the distress)

### Patients’ and family caregivers’ perceived effects of the EPHECT intervention

#### Safe haven

The first visits of the PHC team were mainly used to build up a relationship with the patient and the family caregiver. Although the health condition of most patients was stable when the PHC team first visited them, patients and family caregivers evaluated the visits as positive. They reported in the interviews that having the support of the PHC team whilst receiving chemotherapy resulted in feelings of safety and trust. Especially family caregivers felt safe because they knew who they could turn to when their beloved one’s condition would deteriorate.

*“You’re not alone anymore, it comforts me that I know where I have to go to if I need someone or support.” (family caregiver)*

#### Quality of life

Almost all patients reported that the EPHECT intervention had a positive effect on their quality of life by interventions done or advised by the nurse of the PHC team to relieve discomfort and to optimize their symptom management.

#### Communication between patient and family

Some patients and family caregivers felt that the nurse facilitated conversations, especially when they were not used to talk to their partner or family about their worries. One oncologist said in the interview that the EPHECT intervention was of great value for a patient and her family because it brought the family together and facilitated communication about the dying process.

*“This study really had an effect on that family. Because of your support, they have learned to communicate and they have grown more towards each other.” (oncologist)*

#### Empowerment and advance care planning

Some patients mentioned that the visits of the nurse of the PHC team helped them in reflecting on their wishes and needs and in discussing them with other professional caregivers. Two oncologists also reported that they had the feeling that patients who participated in the EPHECT intervention became more assertive in stating their wishes for future care and communicating their concerns than they were before the start of the intervention.

## Discussion

### Main findings

This study shows that early integration of palliative home care in oncology treatment is feasible and accepted for the most part by patients, family caregivers and professional caregivers. Most of the participating patients, family caregivers and professional caregivers valued the visits of the PHC team. Patients experienced feelings of empowerment, safety and control and reported to have received support to optimize their quality of life. However, important challenges were found with some of the intervention components that had an influence on the feasibility and acceptability of the intervention. Telephone-based contact appeared to be insufficient to support collaboration between disciplines and settings. Furthermore, the GP was not involved more in the care trajectories than before the EPHECT intervention and in most cases he or she had difficulties with practicing the role of coordinator of care.

### Discussion of the main findings

The majority of patients wants to be cared for and die at home [11, 22]. Whilst previous studies focused on integrating palliative care early in a hospital context, the EPHECT intervention aims to introduce palliative care early in the home context increasing the chances of dying at home [23]. Another important added value compared with some of the previous early palliative care interventions [4, 9, 10] is that we performed a phase 2 study focusing on feasibility and acceptability of the intervention components, taking into account the experiences of patients, family caregivers as well as those of professional caregivers involved in the intervention. Performing a phase 2 study is more cost-effective than immediately carrying out a large-scale phase 3 RCT because now we can refine the intervention model based on the experiences of the participants of the phase 2 study [14].

The drop-out of patients due to death during the intervention period is striking given the inclusion criterium of having an estimated life expectancy of 6 to 24 months. All participating oncologists reported that it was possible to estimate a life expectancy of 6 months, but they found a life expectancy of 24 months more difficult to predict. Previous research also shows that it can be difficult for oncologists to estimate survival and that oncologists might have the tendency to be overly optimistic [24–26]. Another possible explanation is that oncologists might have deliberately included patients with a shorter life expectancy out of fear to deprive the hope of patients with a longer estimated survival time, which has been reported as a barrier for early integration of palliative home care in several previous studies [27–33]. Future interventions on early integrated palliative home care should have the discussion of using needs-based or prognosis-based criteria to determine the eligibility of patients.

The protocol of the EPHECT intervention included monthly consultations with a nurse of the palliative home care (PHC) team. However, two thirds of the participants received fewer consultations; the nurse of the PHC team visited the patients on average four times in the 6-month intervention period. According to patients and family caregivers as well as according to the PHC team, systematic visits are important at the beginning of the trajectory because these visits allow to build a relationship of trust. Once the relationship is built, monthly consultations are acceptable to patients but not necessary as long as the patient’s situation is stable. In our intervention protocol, we recommended to plan monthly visits to patients based on previous interventions showing a positive effect of systematic and early integration of palliative care on patient-reported outcomes [3–5, 7, 9, 10, 34]. However, it remains unclear if continuing systematic consultations at home is more effective than follow-up consultations by telephone as used in the ENABLE interventions wherein structured telephone sessions were organized by a PC nurse [3, 4]. Future research is needed to investigate the optimal frequency, structure and implementation of palliative care consultations to improve the quality of life of patients with advanced cancer.

The home visits by the nurse of the PHC team were mainly focused on symptom management and illness understanding, but also psychosocial and existential issues were addressed, which confirms findings of previous intervention studies on early palliative care [35–37]. As expected by the focus groups with PHC team in our previous research [38], psychological issues are more prominent earlier in the disease trajectory. At the beginning of the intervention period the nurses of the PHC team feared that they would not be able to manage these issues because their previous experience was mainly focused on acute symptom management [38]. However, their self-efficacy increased during the intervention. In the information sessions for nurses of the PHC team we did not focus on communication but to be prepared for and feeling safe in having these conversations, we would recommend to take this into account in future trainings for palliative care teams.

This study also found that some family caregivers had the feeling that the visits of the nurses of the PHC team were mainly focused on the patient and that sometimes little attention was given to how family caregivers were coping with the disease of their beloved one. Furthermore, analyses of the HADS questionnaire for family caregivers show that initial scores on anxiety were high and remained high during the intervention period. Looking at previous existing interventions, explicit advise and guidelines on involving the family caregiver were rarely made. In the EPHECT intervention coping of the family caregiver was integrated in the semi-structured conversation guide as a topic needed to be discussed. However, analyses of the logbooks of the team confirm that this topic was the least discussed one during the conversations.

Family caregivers are often defined as unpaid, informal providers of care who have a personal connection to the patient and provide – especially when patients want to be cared for at home – one or more physical, social, practical and emotional tasks. By doing this, they are important actors in providing holistic palliative care [38]. However, taking up these tasks can cause anxiety and other several needs that are often undertreated or not addressed [39–41]. Palliative care teams need to focus more on supporting the family caregiver in caring for their beloved one, as well as taking care of their own needs.

Our intervention results shows that telephone-based contacts mostly failed to improve interdisciplinary and transmural collaboration. A recent influential Commission paper states that a multidisciplinary team approach, with systematic collaboration among team members from different professions within and across levels of care, is needed to strive for optimized integrated palliative care [42]. A systematic review on interventions focusing on integration and oncology care reveals that of the seven included only one advised to routinely involve palliative care teams in multidisciplinary tumor conferences and in only three of them communication and collaboration between the palliative and the oncological service was established [43, 44]. There needs to be a shift from coordinated care in which different teams are linked but working in separate structures to integrated care, in which professionals from different disciplines and settings are gathered together to discuss future care goals. To strive for optimized integrated care, the EPHECT intervention needs to be adapted on interprofessional collaboration. Future trials need to consider using interprofessional collaboration as an important outcome to discuss the effectiveness of an intervention focused on integrated palliative care.

Although we did not choose for a RCT design and hence missed a control group to reflect on the trends in scores on the EORTC QLQ C-30, HADS and FAMCARE, the lack of deterioration in the scores combined with the subjective perceptions of patients and family caregivers showed that the EPHECT intervention had beneficial effects on different domains mentioned in the definition of Heath Related Quality of Life (HRQoL), leading to the assumption that the intervention increased the quality of life of patients and family caregivers. However, given the high scores on of family caregivers on the anxiety subscale of the HADS, it will be important in future interventions on early integrated palliative home care to take into account the mood of family caregivers as an important outcome to make assumptions on the effectiveness of the intervention.

### Strengths and limitations

Performing a phase 2 study is more cost-effective than immediately carrying out a large-scale phase 3 RCT. Based on the results of this phase 2 study necessary adaptations can be made on the intervention model before testing it in a trial with a larger study population. Furthermore, conducting interviews with patients, family caregivers and professional caregivers provided clear insights on the feasibility and acceptability of the EPHECT intervention. However, several limitations exist with regards to the study design. First, by conducting a pre post design, we had no control group. This makes it difficult to make assumptions about the trends seen in patient and family caregiver outcomes. Second, because of the complex nature of the intervention, it is not possible to make conclusions about the effectiveness of individual components. We did not examine how the intervention components were linked and which causal mechanisms could lead to positive effects on certain outcomes. Complementing the study with a more explicit theory-driven approach and a thorough process evaluation could have improved the design and evaluation of this complex intervention [39].

## Conclusion

This study shows that early integration of existing palliative home care in oncology treatment is feasible and accepted for the most part by patients, family caregivers and professional caregivers. By making only small adjustments to current practice, nurses of PHC teams are able to deliver palliative care to advanced cancer patients before they are terminally ill. However, this phase 2 pre-post study also provides essential information on how the EPHECT intervention can be optimized before testing its effectiveness in a large-scale phase 3 RCT. More attention needs to be given to the support of the care giving by family caregivers and to health care provision of family caregivers. Furthermore, the optimal frequency and structure of the home visits by the nurse of the PHC team have to be further evaluated and adapted. More challenging is the optimization of the integration model. To strive for optimized integrated palliative care, professional caregivers from different disciplines and settings have to find a more efficient way to discuss commonly future care goals.

## Data Availability

The data of this study is kept by the first author and is available upon request.

## Abbreviations

PHC: Palliative home care
GP: General practitioner
WHO: World Health Organization
RCT: Randomized controlled trial
